# SynerHyper: Predicting Anticancer Drug Synergy for Lung Cancer Using Uni-Mol Enhanced Hypergraph Neural Networks and Multimodal Fusion

**DOI:** 10.3390/ijms27198500

**Published:** 2026-09-23

**Authors:** Chuance Sun, Linghao Zhang, Xiaotang Huang, Jingyang Li, Jingjing Li, Buyong Ma, Yanjing Wang

**Affiliations:** 1 Engineering Research Center of Cell & Therapeutic Antibody (MOE), Shanghai Frontiers Science Center for Drug Target Identification and Delivery, School of Pharmacy, Shanghai Jiao Tong University, Shanghai 200240, China; sunchuance123@sjtu.edu.cn (C.S.); suspectf@sjtu.edu.cn (L.Z.); lijj@sjtu.edu.cn (J.L.); 2 Zhiyuan College, Shanghai Jiao Tong University, Shanghai 200240, China; real_hhhhhrs@sjtu.edu.cn; 3 School of Life Science and Technology, Harbin Institute of Technology, Harbin 150001, China; doraljy1110@163.com

**Keywords:** drug synergy, drug combination, lung cancer, hypergraph neural network, ensemble learning

## Abstract

Lung cancer remains a leading cause of cancer-related mortality worldwide, and combination therapy is an important strategy for improving treatment efficacy and overcoming drug resistance. However, identifying synergistic drug combinations remains challenging because of the large combinatorial search space and the strong dependence of drug responses on cellular context. Here, we present SynerHyper, a multimodal computational framework for lung cancer drug synergy prediction that integrates three-dimensional molecular representations, transcriptomic profiles, hypergraph learning, and ensemble fusion. Drug molecules are encoded using a Uni-Mol-based molecular encoder, while high-dimensional cell-line gene-expression profiles are compressed using a deep autoencoder. Each drug–drug–cell-line observation is represented as a three-node hyperedge, enabling a two-layer hypergraph neural network to model higher-order interactions among the two drugs and their cellular context. Data augmentation is applied exclusively to the training subset to increase training-data diversity and mitigate class imbalance, and predictions from the hypergraph model, multilayer perceptron, and LightGBM are combined through soft ensemble fusion. Under the split-before-augmentation evaluation protocol, the validation set was used for model selection, while the test set remained completely isolated until final evaluation. SynerHyper achieved a validation AUC of 0.815 and an independent test AUC of 0.808, outperforming the strongest baseline DeepDDS (GCN, 0.792) and other representative methods including AttenSyn (0.780), DeepSynergy (0.750), and DTSyn (0.690) under the same lung cancer dataset. Together with an accessible online prediction server (SynerHyper), SynerHyper provides a lung cancer-specific computational framework for prioritizing candidate drug combinations for downstream experimental validation.

## 1. Introduction

Combination therapy is a central strategy in modern oncology, as simultaneous perturbation of multiple biological pathways can enhance therapeutic efficacy, delay the emergence of drug resistance, and potentially reduce the required dose of individual agents [1,2,3,4]. Despite its clinical potential, identifying synergistic drug combinations remains challenging, particularly for lung cancer, one of the most common and molecularly heterogeneous malignancies [5,6]. The number of possible drug pairs increases quadratically with the number of candidate compounds, while the biological response to a given combination depends strongly on cancer type, cell-line-specific molecular context, and drug mechanism of action. Therefore, computational approaches capable of prioritizing promising drug combinations for lung cancer before experimental validation are highly desirable.

Recent advances in computational drug synergy prediction have led to the development of numerous machine-learning and deep-learning frameworks, including SynergyX [7], VQSynergy [8], LGSyn [9], DDSBC [10], HypergraphSynergy [11], DVHSyn [12], HERMES [13], and HIG-Syn [14]. SynergyX employs multimodal mutual attention to model cross-modal drug–cell interactions; VQSynergy combines vector quantization with hypergraph convolution for representation learning; LGSyn integrates local molecular features with global knowledge-graph information; DDSBC adopts a stacking ensemble strategy for cancer-specific synergy classification; HypergraphSynergy models multi-way drug–drug–cell-line relationships using hypergraphs; DVHSyn introduces dual-view hypergraph fusion to capture complementary relational information; HERMES incorporates disease indications and gated residual hypergraph networks to improve representation learning and support prediction involving unseen drugs; and HIG-Syn combines multigranularity hypergraph modeling with interaction-aware attention to characterize both global triplet relationships and fine-grained drug–cell-line interactions. Collectively, these approaches integrate molecular fingerprints, molecular graphs, transcriptomic profiles, protein–protein interaction networks, knowledge graphs, hypergraphs, and attention-based fusion to model drug-combination responses. However, most existing methods are developed as general or pan-cancer frameworks using datasets spanning multiple tumor types and biological contexts. Such models may not fully capture lung cancer-specific molecular heterogeneity and cell-state-dependent drug responses. In addition, some existing approaches rely predominantly on two-dimensional molecular representations or pairwise interaction modeling, which may provide limited representation of three-dimensional molecular structure and higher-order relationships among drug combinations and cellular contexts.

Drug synergy is inherently dependent on cellular context. The same drug pair may exhibit synergistic, additive, or antagonistic responses across different cell lines because of differences in gene expression, pathway activity, genetic background, and cellular dependencies. Therefore, the fundamental observation in a drug-combination screen is not simply a drug–drug pair, but a drug A–drug B–cell-line triplet. Conventional pairwise graphs typically represent drug–drug and drug–cell-line relationships using separate edges and may not explicitly preserve the complete experimental context of an observed combination response. In contrast, a hypergraph allows each measured drug-combination response to be represented as a single hyperedge connecting the two compounds and the corresponding cell line, thereby preserving the higher-order contextual relationship among all three entities. To address these challenges, we developed SynerHyper, a lung cancer-specific multimodal framework for drug synergy prediction. SynerHyper integrates Uni-Mol-derived molecular embeddings with transcriptomic representations of lung cancer cell lines, thereby incorporating both drug structural information and cellular context [15]. Each drug–drug–cell-line triplet is represented as a hyperedge, enabling the model to capture higher-order relationships that are not explicitly represented by conventional pairwise graph structures. Training-data augmentation based on drug-pair symmetry and feature-level perturbation is used to increase feature diversity and mitigate class imbalance. Predictions from the hypergraph neural network are further integrated with multilayer perceptron and LightGBM models through soft ensemble fusion [16,17,18].

The main contributions of this study are as follows. First, we develop a lung cancer-specific multimodal framework that integrates three-dimensional molecular representations with transcriptomic cell-line features. Second, we formulate drug synergy prediction as a higher-order hypergraph learning problem in which each drug–drug–cell-line observation is represented as a single hyperedge. Third, we introduce training-set-specific data augmentation based on drug-pair symmetry and feature-level perturbation to increase training diversity and mitigate class imbalance. Fourth, we integrate hypergraph learning with MLP and LightGBM models through soft ensemble fusion to exploit complementary predictive information. Fifth, we evaluate the framework using a strict split-before-augmentation protocol in which preprocessing, representation learning, augmentation, and model optimization are restricted to the training data, while the independent test set remains isolated until final evaluation. Finally, we provide downstream analyses including cell-line-specific performance evaluation, embedding visualization, retrospective assessment of prioritized drug combinations, and an online prediction server for experimental prioritization.

## 2. Results

### 2.1. Autoencoder Reconstruction Performance

To obtain compact representations of high-dimensional transcriptomic profiles while avoiding information leakage during representation learning, the autoencoder-based feature compression module was fitted exclusively using data from the training subset. The original 4079-dimensional gene-expression profiles were compressed into 512-dimensional latent representations using mean squared error (MSE) reconstruction loss. As shown in Figure 1, the reconstruction loss decreased rapidly during the early stage of training and gradually stabilized as optimization proceeded, indicating stable convergence of the autoencoder. After training, the encoder parameters were fixed and the learned transformation was subsequently applied to the validation and test subsets without further parameter updating. Thus, the transcriptomic representations used in downstream prediction were generated using a feature transformation learned exclusively from the training data. The resulting 512-dimensional latent embeddings were then integrated with drug molecular representations for subsequent multimodal and hypergraph-based drug synergy prediction.

### 2.2. Predictive Performance Under the Split-Before-Augmentation Evaluation Protocol

To obtain a more rigorous estimate of predictive performance and prevent augmentation-induced information leakage, the original dataset was first divided into training, validation, and test subsets before any data augmentation or model fitting. All preprocessing procedures requiring parameter estimation, including feature scaling and autoencoder training, were fitted exclusively using the training subset, and data augmentation was restricted to the training data. The validation subset was used for model selection and parameter optimization, whereas the test subset was excluded from model fitting, preprocessing fitting, augmentation, hyperparameter tuning, ensemble-weight optimization, and checkpoint selection.

During model training, the training and validation losses were monitored across epochs to characterize optimization behavior and model convergence (Figure 2A). In parallel, predictions from the hypergraph, MLP, and LightGBM components were combined through soft ensemble fusion using weights of 0.4, 0.3, and 0.3, respectively. The resulting ensemble AUC and F1 scores were recorded for the training, validation, and test subsets across epochs (Figure 2B,C). Importantly, test-set metrics were monitored for descriptive purposes only and were not used to update model parameters, optimize ensemble weights, select checkpoints, tune hyperparameters, or make any other model-selection decisions. The final checkpoint was selected exclusively according to validation-set performance.

At the validation-selected checkpoint, the SynerHyper ensemble achieved a validation AUC of 0.815 and a corresponding test AUC of 0.808, indicating that the predictive performance observed during model development was largely retained on the held-out test subset. The corresponding test-set confusion matrix showed 1878 true negatives, 197 false positives, 221 false negatives, and 817 true positives (Figure 2D), yielding a precision of approximately 0.806, a recall of 0.787, and an F1 score of 0.796. The prediction-score distributions further showed that synergistic samples generally received higher predicted probabilities than non-synergistic samples, while the overlap between the two classes remained around the decision boundary (Figure 2E). Overall, these results demonstrate that SynerHyper maintained stable and competitive predictive performance under the revised split-before-augmentation evaluation protocol.

### 2.3. Comparison with State-of-the-Art Drug Synergy Prediction Methods

To evaluate the performance of SynerHyper in the lung cancer-specific setting, we compared it with several representative drug-synergy prediction models, including DTSyn, DeepSynergy, DeepDDS (GAT/GCN), and AttenSyn. Importantly, the AUC values reported in Table 1 were generated in the present study rather than taken from the original publications. The corresponding model implementations were applied to the lung cancer prediction dataset, and their predictive performance was evaluated using the resulting predictions. These methods were originally developed as general anticancer drug-synergy prediction frameworks incorporating drug and cancer-cell-line information and were therefore applicable to the lung cancer samples considered here. However, they were not specifically retrained or optimized for the lung cancer setting; thus, the comparison primarily assesses their transfer performance from general drug-synergy modeling to lung cancer-specific prediction.

As shown in Table 1, SynerHyper achieved the highest test AUC of 0.808 among the evaluated methods. DeepDDS (GCN) showed the strongest baseline performance with an AUC of 0.792, followed by AttenSyn (0.780), DeepSynergy (0.750), DeepDDS (GAT) (0.714), and DTSyn (0.690). Thus, SynerHyper improved the test AUC by 0.016 relative to the strongest competing baseline. Although the improvement was moderate, the results indicate that SynerHyper maintained competitive predictive performance under the revised split-before-augmentation evaluation protocol. The observed performance may reflect the combined contribution of lung cancer-specific transcriptomic representations, Uni-Mol-derived molecular features, higher-order drug–drug–cell-line modeling, and ensemble fusion. Nevertheless, the present comparison should be interpreted as a performance comparison within the current lung cancer dataset rather than as definitive evidence of architectural superiority across all drug-synergy prediction settings. Therefore, these results suggest that disease-specific modeling may provide advantages when prioritizing drug combinations within a defined cancer context, although the comparison should not be interpreted as a controlled assessment of architectural superiority under identical model-training procedures.

### 2.4. Ablation Study

To investigate the contribution of individual components within SynerHyper, we conducted a series of ablation experiments under the same revised split-before-augmentation evaluation protocol. As shown in Table 2, the complete framework achieved a test AUC of 0.808, outperforming all ablated variants.

Removing the hypergraph branch resulted in a marked decrease in performance, with the test AUC dropping from 0.808 to 0.722, indicating that explicit modeling of higher-order drug–drug–cell-line relationships makes an important contribution to the overall framework. Replacing Uni-Mol-based molecular representations with traditional molecular fingerprints produced the largest performance reduction, decreasing the AUC to 0.716, which supports the value of incorporating structurally informed molecular representations. Similarly, removing the autoencoder-based transcriptomic compression module reduced the AUC to 0.736, suggesting that compact latent representations of cell-line transcriptomic profiles improve downstream prediction compared with using raw transcriptomic features.

The MLP branch and ensemble fusion also contributed to the final performance. Removing the MLP branch reduced the AUC to 0.780, while replacing soft ensemble fusion with direct feature concatenation resulted in an AUC of 0.792. Although these reductions were smaller than those observed after removing the hypergraph, Uni-Mol, or autoencoder components, they indicate that nonlinear feature learning and prediction-level integration provide complementary information to the overall framework.

Consistent with these observations, the hypergraph-only model achieved an AUC of 0.773, whereas the MLP-only model achieved an AUC of 0.668. The higher performance of the hypergraph-only branch further supports the importance of explicitly modeling higher-order relational structure. Nevertheless, neither individual branch matched the performance of the complete framework, indicating that the final predictive performance results from the combined contributions of molecular representation learning, transcriptomic compression, hypergraph modeling, and ensemble integration.

### 2.5. Cell-Line-Specific Performance Analysis

To further characterize the predictive performance of SynerHyper across different lung cancer cellular contexts, we evaluated the ensemble model separately on six representative lung cancer cell lines within the held-out test subset. The number of test samples per cell line ranged from 456 to 495, and multiple classification metrics, including AUC, average precision (AP), F1 score, recall, and precision, were calculated to provide a comprehensive assessment of cell-line-specific performance. As shown in Figure 3A, the test AUC values ranged from 0.759 to 0.842, indicating generally stable but cell-line-dependent discriminative performance. Among the six evaluated cell lines, HOP-92 showed the highest AUC (0.842), followed by NCI-H522 (0.828) and NCI-H460 (0.812). In contrast, HOP-62 exhibited the lowest AUC (0.759), followed by EKVX (0.769). These differences suggest that the predictive difficulty of drug synergy varies across cellular backgrounds even under the same evaluation protocol.

The F1-score analysis showed a similar pattern (Figure 3B). F1 scores ranged from 0.750 to 0.812, with HOP-92 again achieving the highest value (0.812) and HOP-62 the lowest (0.750). The comprehensive heatmap further demonstrated corresponding variation in recall, precision, and average precision across cell lines (Figure 3C). Recall ranged from 0.738 to 0.801, precision from 0.764 to 0.826, and AP from 0.771 to 0.849. HOP-92 achieved the highest values across all three metrics, whereas HOP-62 generally showed the lowest performance. Overall, the cell-line-specific results indicate that SynerHyper maintained useful predictive performance across multiple lung cancer cellular contexts, while also revealing measurable heterogeneity among individual cell lines.

Importantly, these results represent subgroup analyses within the held-out test subset rather than a strict evaluation of generalization to completely unseen cell lines. Because the same cell-line identities may be represented in both the training and test partitions, the present analysis assesses predictive consistency across known cellular contexts rather than leave-cell-line-out generalization. Therefore, the observed differences should be interpreted primarily as cell-line-dependent variation in predictive performance within the current dataset.

### 2.6. Visualization and Quantitative Assessment of Learned Hypergraph Embeddings

To evaluate changes in the representation space after model learning, we compared the raw multimodal features with the learned embeddings using principal component analysis (PCA), t-distributed stochastic neighbor embedding (t-SNE), and multiple quantitative class-separation metrics.

As shown in Figure 4A,C, synergistic and non-synergistic samples were extensively intermixed in the raw feature space, with substantial overlap between the two classes. After representation learning, the PCA and t-SNE projections showed improved local organization of the learned embeddings (Figure 4B,D). The class centroids became more distinguishable and the corresponding class distributions exhibited greater directional separation, although considerable overlap between synergistic and non-synergistic samples remained. Therefore, the low-dimensional projections indicate improved organization of the learned feature space rather than complete class separation.

To complement the visual assessment, we further quantified class separation using six complementary metrics (Figure 4E). The silhouette score increased from 0.0079 for the raw features to 0.0127 for the learned embeddings, while the Calinski–Harabasz index increased from 35.04 to 187.58. Conversely, the Davies–Bouldin index decreased from 30.91 to 14.89, consistent with improved class compactness and relative separation. The Mahalanobis distance between classes increased from 0.690 to 0.778, and the Fisher discriminant ratio increased from 4.85 to 6.52, indicating greater separation between the class distributions relative to their within-class variation. In addition, the inter-/intra-class distance ratio increased from 0.00077 to 0.00442. Collectively, all six quantitative metrics changed in the direction expected for improved class organization after representation learning.

Nevertheless, the absolute silhouette score and inter-/intra-class distance ratio remained low, indicating that the learned representations did not form clearly separated clusters. This is consistent with the substantial overlap still visible in the PCA and t-SNE projections and reflects the heterogeneous and context-dependent nature of anticancer drug synergy. Accordingly, the learned embeddings should be interpreted as showing a modest but consistent improvement in discriminative organization rather than strong or complete class separation.

### 2.7. Retrospective Support for Selected High-Ranking Drug Combinations

We further examined high-ranking candidate synergistic drug combinations that were absent from the training subset and retrospectively assessed their pharmacological plausibility through literature mining and public drug synergy databases.

Among the 18 high-ranking predicted drug combinations, 8 pairs (44.4%) were supported by previously published experimental studies, while 5 additional pairs (27.8%) were supported by the O’Neil 2016 drug synergy dataset (Figure 5A). The remaining 5 predicted pairs (27.8%) lacked currently available supporting evidence, indicating that these combinations represent potential hypotheses requiring further experimental validation. Overall, 13 of the 18 predicted combinations (72.2%) were supported by either published literature or held-out public screening data, suggesting that SynerHyper is capable of identifying pharmacologically plausible synergistic interactions. Analysis of evidence sources revealed that literature-supported combinations were derived from diverse experimental contexts, including cell-based synergy assays, mechanistic studies, and preclinical investigations (Figure 5B). In parallel, several predicted interactions were also identified in the held-out O’Neil 2016 drug synergy dataset, providing additional retrospective support from systematic drug combination screening. The evidence heatmap showed that supported predictions were distributed across multiple drug classes, including kinase inhibitors, microtubule-targeting agents, DNA-damaging drugs, and other anticancer compounds, rather than being restricted to a single therapeutic category (Figure 5C).

Mechanistic network analysis further suggested that the supported drug pairs formed biologically interpretable interaction modules (Figure 5D). Several combinations involved complementary regulation of key cancer-associated processes, including cell-cycle checkpoint control, receptor signaling pathways, DNA damage response, and microtubule dynamics. For example, MK-1775 (Adavosertib), a WEE1 inhibitor, showed synergistic associations with agents involved in cell-cycle regulation and DNA damage-related mechanisms, consistent with the role of checkpoint inhibition in enhancing tumor cell vulnerability. Similarly, Erlotinib-based combinations were linked to EGFR-associated signaling inhibition and PARP-related therapeutic mechanisms, whereas Vinorelbine- and Vincristine-centered combinations were primarily associated with microtubule-targeting anticancer strategies.

Collectively, the high proportion of literature- and database-supported predictions provides retrospective evidence for the pharmacological plausibility of the prioritized combinations. However, because the current evaluation does not employ a strict drug- or combination-disjoint splitting protocol, these results should not be interpreted as formal evidence of extrapolation to completely unseen compounds or drug combinations.

### 2.8. Online Web Server for SynerHyper

To improve the accessibility and practical usability of SynerHyper, we further developed an online web server (malab.sjtu.edu.cn/SynerHyper) with an interactive user interface for lung cancer drug synergy prediction (Figure 6). The platform allows users to conveniently input candidate drug combinations and perform real-time synergy prediction without requiring local model deployment or programming expertise. The web server integrates the trained SynerHyper framework, including transcriptomic representation learning, hypergraph-based interaction modeling, and ensemble prediction modules. Users can select drug pairs through the prediction interface and obtain predicted synergy scores for downstream analysis. In addition, the platform provides model highlights, visualization components, and user-friendly navigation modules to facilitate practical application and experimental prioritization. Overall, the online server extends the usability of SynerHyper and provides an accessible tool for researchers investigating synergistic drug combinations in lung cancer. The web server is intended solely as a research-oriented prioritization tool. Its predictions should not be interpreted as clinically validated therapeutic recommendations, and candidate combinations require independent experimental confirmation before biological or translational application.

## 3. Discussion

In this study, we developed SynerHyper, a Uni-Mol-enhanced multimodal hypergraph framework for lung cancer drug synergy prediction. Under the revised split-before-augmentation evaluation protocol, SynerHyper achieved a test AUC of 0.808, showing competitive predictive performance compared with representative drug-synergy prediction models evaluated on the same lung cancer dataset. In particular, SynerHyper achieved a higher test AUC than DeepDDS (GCN) (0.792), AttenSyn (0.780), DeepSynergy (0.750), DeepDDS (GAT) (0.714), and DTSyn (0.690). Cell-line-specific analyses further showed test AUC values ranging from 0.759 to 0.842 across six lung cancer cell lines, suggesting that the framework retained useful predictive capability across multiple cellular contexts, although measurable cell-line-dependent heterogeneity remained. The proposed framework integrates molecular structural information, transcriptomic cell-line profiles, higher-order hypergraph modeling, training-set-specific data augmentation, and ensemble learning. Unlike conventional pairwise formulations, SynerHyper explicitly represents each drug–drug–cell-line observation as a hyperedge, thereby preserving the higher-order relationship between the two compounds and their cellular context.

The ablation experiments further supported the contribution of the major components of the framework. Replacing Uni-Mol representations with traditional molecular fingerprints reduced the test AUC from 0.808 to 0.716, while removing the hypergraph branch reduced it to 0.722. Removing the autoencoder-based transcriptomic compression module also decreased performance to 0.736. These results suggest that structurally informed molecular representations, higher-order relational modeling, and compact transcriptomic features each contribute to the overall predictive performance. Removing the MLP branch produced a more moderate reduction in AUC to 0.780, whereas replacing soft ensemble fusion with direct concatenation resulted in an AUC of 0.792, indicating that complementary feature learning and prediction-level integration provide additional, although smaller, benefits. Taken together, these findings suggest that the performance of SynerHyper arises from the combined contribution of multiple representation and modeling components rather than from a single module.

The representation-space analyses provided additional insight into how model learning altered the organization of the multimodal features. Compared with the raw feature space, the learned embeddings showed improvements across all six quantitative class-separation metrics. The Calinski–Harabasz index increased from 35.04 to 187.58, the Davies–Bouldin index decreased from 30.91 to 14.89, the Mahalanobis distance increased from 0.690 to 0.778, and the Fisher discriminant ratio increased from 4.85 to 6.52. However, the silhouette score remained low (0.0079 to 0.0127), and substantial overlap between synergistic and non-synergistic samples remained visible in both PCA and t-SNE projections. Therefore, these results should be interpreted as evidence of modest improvement in discriminative organization rather than formation of clearly separated biological clusters. This observation is consistent with the heterogeneous and context-dependent nature of anticancer drug synergy.

Several limitations should still be considered when interpreting the present results. First, although the revised evaluation pipeline reduces the risk of augmentation-related information overlap by performing dataset partitioning before augmentation by performing dataset partitioning before preprocessing and augmentation, the current split remains sample-based. Individual drugs, drug pairs, or cell-line identities may therefore occur in more than one subset. Accordingly, the present results do not establish strict inductive generalization to completely unseen drugs, drug combinations, or cell lines. Future studies should therefore include leave-combination-out, leave-drug-out, and leave-cell-line-out evaluations. Second, the integrated public datasets were generated using different experimental platforms, concentration ranges, synergy definitions, and quality-control procedures, and residual study-specific heterogeneity may remain after preprocessing. Third, the current framework uses binary synergy labels and baseline transcriptomic profiles and does not explicitly incorporate dose dependence, mutations, copy-number alterations, proteomics, drug targets, or pathway activity. Incorporating such information may provide a more comprehensive characterization of context-dependent drug responses. Fourth, although retrospective literature and O’Neil database evidence supports the pharmacological plausibility of several prioritized drug combinations, such evidence does not constitute prospective experimental validation. All candidate combinations generated by SynerHyper therefore require confirmation using dose–response matrices and appropriate in vitro or in vivo models before biological or translational interpretation.

## 4. Materials and Methods

### 4.1. Overview of the SynerHyper Framework

The proposed SynerHyper framework consists of five major components (Figure 7a): data preprocessing, molecular representation learning, cell-line feature compression, hypergraph construction and learning, and ensemble prediction. After deterministic data curation and filtering, the dataset was divided into training, validation, and test subsets before any fitted preprocessing or data augmentation. All parameter-dependent preprocessing procedures, including feature scaling and autoencoder training, were fitted exclusively using the training subset, and data augmentation was applied only to the training data.

**Figure 7:**
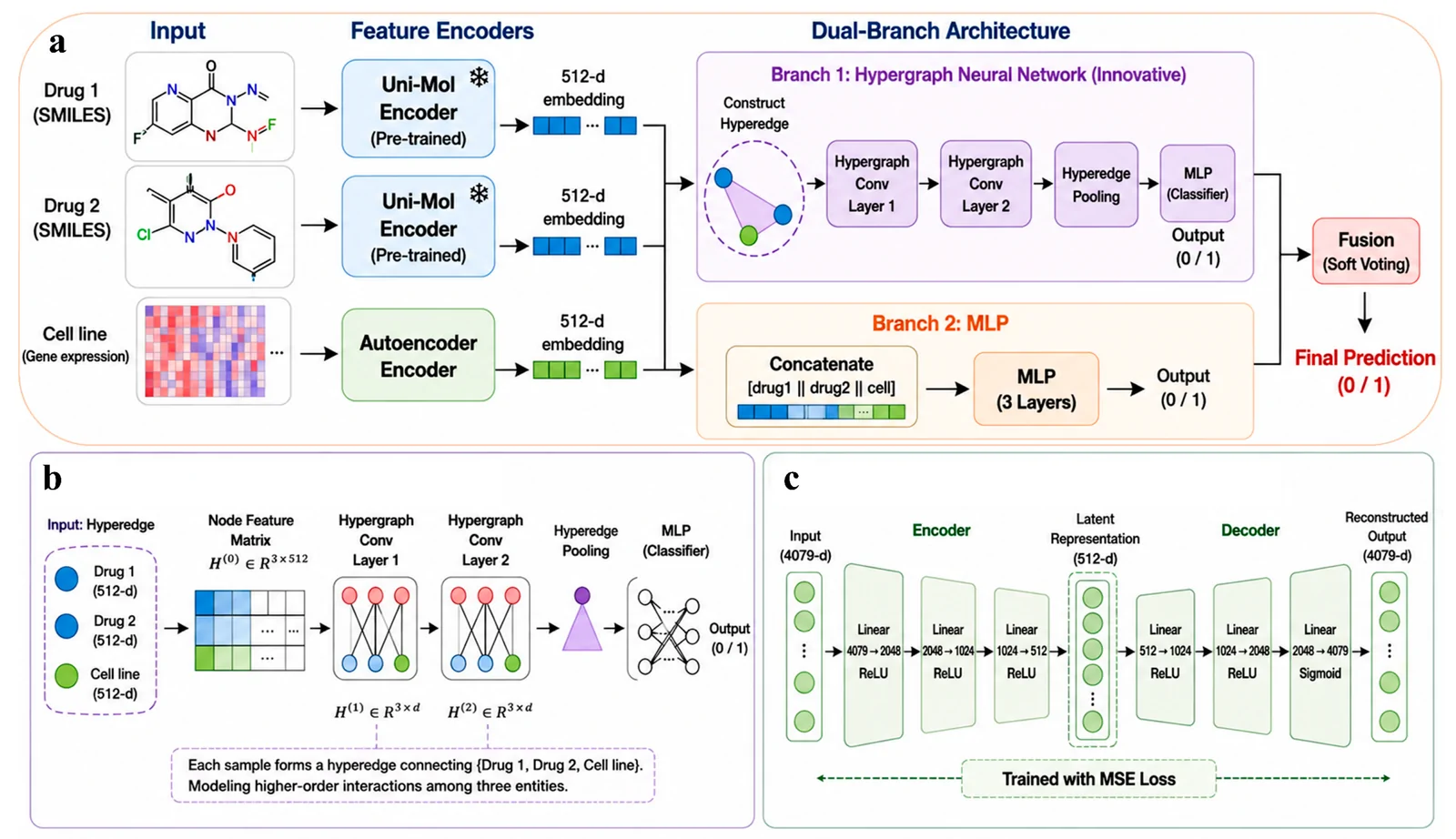
The framework of SynerHyper. (**a**) Overall pipeline of the proposed framework. Drug molecules are encoded into 512-dimensional representations using a Uni-Mol-based encoder that incorporates three-dimensional structural information. Lung cancer cell-line transcriptomic profiles (4079 genes) are compressed into a 512-dimensional latent space via an autoencoder. A dual-branch architecture is adopted: a hypergraph neural network (HGN) branch models higher-order drug–drug–cell-line interactions, while an MLP branch learns nonlinear patterns from concatenated drug–drug-cell-line features. Final predictions are obtained through soft ensemble fusion. (**b**) Hypergraph construction and learning process. Each drug combination under a specific cell line is modeled as a hyperedge connecting three nodes (Drug 1, Drug 2, Cell line). Node features are propagated through stacked hypergraph convolution layers, followed by hyperedge-level pooling and classification. This formulation explicitly captures higher-order interactions beyond pairwise relationships. (**c**) Autoencoder-based cell-line feature compression. The encoder compresses high-dimensional (4079-dim) gene expression profiles into a 512-dimensional latent representation, while the decoder reconstructs the original input from this compressed representation. The model is optimized using mean squared reconstruction loss, and the learned latent vectors are used as compact cell-line embeddings for downstream prediction.

Drug molecules were encoded into 512-dimensional representations using a Uni-Mol-based molecular feature extractor, while high-dimensional cell-line gene-expression profiles were compressed into 512-dimensional latent representations using an autoencoder trained on the training data. Each drug–drug–cell-line observation was represented as a hyperedge connecting drug 1, drug 2, and the corresponding cell line, and a hypergraph neural network was trained to classify drug synergy. In parallel, MLP and LightGBM models were trained using concatenated multimodal features. Finally, predictions from the hypergraph model, MLP, and LightGBM were combined through soft ensemble fusion to obtain the final synergy score.

### 4.2. Drug Synergy and Cell-Line Datasets

The drug synergy prediction task uses two large public datasets: DrugComb (accessed on October 2024) and GDSC2 (accessed on December 2024). The DrugComb dataset (available at http://drugcombdb.denglab.org/main, accessed on 17 September 2026) includes data from various screening studies, such as NCI-ALMANAC, O’Neil, FORCINA and CLOUD datasets [19,20,21]. It comprises 8397 drugs, 2320 cell lines, and 33 tissue types, yielding 739,964 drug combinations. The GDSC2 dataset (available at https://gdsc-combinations.depmap.sanger.ac.uk/ accessed on 17 September 2026) is the largest resource for cancer drug combination screening to date [22]. It comprises 109 drug combinations from AstraZeneca’s oncology portfolio, tested across 755 pan-cancer cell lines from 41 cancer types, with 68,816 distinct drug–drug–cell line combinations.

Given the lung cancer-specific focus of this study, only records associated with lung cancer cell lines were retained from the integrated DrugComb and GDSC2 input table. All records originating from the O’Neil study were removed before model development and were reserved only for subsequent supporting analysis. Drug names, cell-line names, SMILES strings, and binary classification labels were read from the curated input table. The current framework used the supplied binary labels directly and did not recalculate synergy scores from raw dose–response matrices. Cell-line identifiers in both the drug-combination table and transcriptomic feature matrix were stripped of leading and trailing spaces before matching. A record was retained only when both drug representations could be generated, the corresponding cell-line transcriptomic profile was available, and the drug and cell-line feature dimensions were mutually compatible. Samples with unmatched cell-line identifiers, missing drug features, invalid molecular representations, or inconsistent feature dimensions were excluded. The current training pipeline did not perform an additional model-specific reconciliation of duplicate or conflicting labels beyond the upstream construction of the curated input table; therefore, possible source-level redundancy and cross-study label heterogeneity remain limitations of the present dataset.

The original dataset contained 31,652 drug combination observations, covering 168 unique small-molecule drugs and 27 cancer cell lines. After preprocessing, 531 observations were removed because of unmatched cell-line identifiers or unavailable compatible features, resulting in 31,121 valid drug–drug–cell-line observations and a sample retention rate of 98.32%. Among these observations, 20,447 were labeled as non-synergistic and 10,674 as synergistic, corresponding to an initial positive-to-negative ratio of approximately 1:1.92. After data curation, the 31,121 valid observations were divided into training, validation, and test subsets before any fitted preprocessing or data augmentation. The resulting subsets contained 21,784 training observations, 6224 validation observations, and 3113 test observations, corresponding approximately to a 70%/20%/10% partition. Data augmentation was subsequently applied exclusively to the training subset, increasing the number of training observations from 21,784 to 36,411, whereas the validation and test subsets remained unchanged. For downstream hypergraph indexing, the augmented training subset together with the unaugmented validation and test subsets comprised 45,748 hyperedges. Following cell-line feature matching, the resulting hypergraph involved 193 nodes, including 168 drugs and 25 matched lung cancer cell lines.

### 4.3. Data Augmentation

Five feature-level augmentation operations were implemented exclusively within the training subset. First, drug-pair symmetry exchanged the representations and identities of drug 1 and drug 2 while retaining the corresponding cell-line representation and class label. Second, Gaussian feature perturbation added zero-mean noise with a standard deviation equal to 5% of the within-vector feature standard deviation. Third, feature dropout randomly set 10% of the dimensions in the drug and cell-line representations to zero. Fourth, feature exchange replaced 20% of the feature dimensions using another observation drawn from the same class. Fifth, cell-line feature mixing linearly combined the cell-line representations of two same-class observations using a fixed mixing coefficient of 0.5. The augmentation strategy was randomly selected for each generated observation, and negative samples were preferentially augmented to mitigate class imbalance.

Importantly, dataset partitioning was performed before any data augmentation. The 31,121 valid observations were first divided into training, validation, and test subsets containing 21,784, 6224, and 3113 observations, respectively. Data augmentation was subsequently applied only to the training subset, increasing its size from 21,784 to 36,411 observations, whereas the validation and test subsets remained unaugmented. The resulting downstream dataset therefore contained 45,748 hyperedges, comprising the augmented training subset together with the original validation and test subsets. Because augmentation was restricted to observations assigned to the training subset after partitioning, augmentation-generated variants of validation or test observations were not introduced into the training data. This split-before-augmentation procedure was adopted to prevent augmentation-induced information overlap across the training, validation, and test partitions.

### 4.4. Molecular Representation Using Uni-Mol

The molecular encoder consisted of 15 transformer-like layers with eight-head self-attention and was initialized using compatible parameters mapped from the pretrained checkpoint unimol_weight.pt. During model initialization, 154 of 211 pretrained parameter groups were successfully mapped. The encoder was used in evaluation mode, and molecular embeddings were generated without gradient updating during downstream synergy-model training. Three-dimensional conformers were generated using RDKit ETKDGv3 with a fixed random seed of 42, followed by MMFF optimization when successful. Among the 168 unique compounds, Uni-Mol-based representations were successfully generated for 167 compounds. One compound failed during conformer processing and was therefore represented using a 512-dimensional fallback vector composed of RDKit physicochemical descriptors and Morgan fingerprints. No compounds were excluded because of missing SMILES information.

$$Attention\left(Q,K,V\right)=softmax(\frac{QK^{T}}{\sqrt{d_{k}}}+M)V$$

where M encodes pairwise distance information between atoms. The final molecular representation is obtained through attention-weighted pooling over atom-level hidden states. Let $h_{i}^{\left(L\right)}$ be the hidden state of atom i after L encoder layers (L=15); the drug embedding $z_{drug}$ is computed as:

$$z_{drug}=\sum_{i=1}^{n}\alpha_{i}h_{i}^{\left(L\right)},\;\;\alpha_{i}=\frac{exp(w^{T}h_{i}^{\left(L\right)})}{\sum_{j=1}^{n}exp(w^{T}h_{j}^{\left(L\right)})}$$

where w is a learnable attention vector. If Uni-Mol processing fails for a given molecule, the framework falls back to traditional RDKit-derived molecular descriptors and Morgan fingerprints, padded or constructed to match a 512-dimensional representation.

### 4.5. Cell-Line Representation Using Autoencoder-Based Dimensionality Reduction

The transcriptomic feature library contained 135 cell-line profiles, each represented by 4079 gene-expression features. Before downstream model training, gene-expression features were standardized, and all transformation parameters were estimated using the training subset only. To avoid information leakage, the autoencoder was trained exclusively using cell-line profiles available in the training subset. The learned encoder was subsequently applied to validation and test samples to generate corresponding 512-dimensional latent representations. The encoder architecture is:

4079→2048→1024→512,

and the decoder mirrors the structure:

512→1024→2048→4079.

Each hidden layer uses batch normalization, ReLU activation, and dropout. The autoencoder is trained using mean squared reconstruction loss:

LAE=1NC∑i=1Ncxci−x^ci2^2

where $x_{c}^{\left(i\right)}\in R^{4079}$ is the original expression profile of cell line i, and ${\hat{x}}_{c}^{\left(i\right)}$ is its reconstruction. The Adam optimizer is used with a learning rate of $1\times 10^{-3}$ for 200 epochs. A ReduceLROnPlateau scheduler adapts the learning rate based on validation loss. After training, the encoder output $z_{c}=f_{\phi }(x_{c})\in R^{512}$ is used as the cell-line representation. The latent features are further standardized before being used in the drug synergy prediction model. Validation reconstruction loss was monitored only for convergence assessment and was not used for gradient updates or early stopping. The encoder parameters used for downstream prediction were those obtained at the end of the fixed 200-epoch training schedule.

### 4.6. Hypergraph Construction

Drug synergy prediction is formulated as a hypergraph classification problem. In the constructed hypergraph G=(V,E), each unique drug and each unique cell line is treated as a node. Each drug-combination sample corresponds to a hyperedge connecting three nodes:

$$e_{ijk}=\left\{d_{i},d_{j},c_{k}\right\}$$

Compared with ordinary graphs that primarily model pairwise edges, this hypergraph representation directly captures the higher-order interaction among two drugs and a cellular context. Node features are assigned according to drug embeddings or cell-line latent vectors. For each hyperedge, the corresponding label indicates whether the drug pair is synergistic in that cell line.

### 4.7. Hypergraph Neural Network

The hypergraph neural network consists of two hypergraph convolution layers followed by a classifier. Define the incidence matrix $H\in R^{|V|\times |E|}$:

H(v,e)=0, otherwise

H(v,e)=1,if  v ϵ e

Let $D_{v}\in R^{|V|\times |V|}$ be the diagonal node degree matrix with $D_{v}(v,v)=\sum_{e\in E}H(v,e)$, and $D_{e}\in R^{|E|\times |E|}$ the diagonal hyperedge degree matrix with $D_{e}(e,e)=\sum_{v\in V}H(v,e)$. The hypergraph convolution operation is defined as:

$$X^{\left(l+1\right)}=\sigma (D_{v}^{-1/2}HD_{e}^{-1}H^{T}D_{v}^{-1/2}X^{\left(l\right)}\Theta^{\left(l\right)})$$

where $X^{\left(l\right)}\in R^{|V|\times d_{l}}$ is the node feature matrix at layer l, $\Theta^{\left(l\right)}\in R^{d_{l}\times d_{l+1}}$ is a learnable weight matrix, and σ(⋅) is the ReLU activation function. In our implementation, $d_{0}=512$, $d_{1}=256$, and $d_{2}=128$. Degree normalization is performed using vectors rather than explicit large diagonal matrices to reduce GPU memory usage. After two convolution layers, the representation of each hyperedge $e=\{d_{i},d_{j},c_{k}\}$ is obtained by averaging the embeddings of its member nodes:

$$h_{e}=\frac{1}{3}(x_{d_{i}}^{\left(2\right)}+x_{d_{j}}^{\left(2\right)}+x_{c_{k}}^{\left(2\right)})$$

The hyperedge representation is then passed through a classifier:

$$\hat{y_{e}}=sigmoid(W_{2}\cdot ReLU\left(W_{1}h_{e}+b_{1}\right)+b_{2})$$

where $W_{1}\in R^{64\times 128}$, $W_{2}\in R^{1\times 64}$, and $b_{1},b_{2}$ are bias terms. A learnable threshold parameter τ∈[0,1] is introduced to enhance negative sample discrimination. For predicted probabilities below τ, an additional transformation is applied:

$${\hat{y}}_{e}^{\left(adj\right)}=sigmoid\left(ReLU\left(h_{e}^{T}u\right)\cdot v\right),\;if\;\hat{y_{e}}<\;\tau$$

where u and v are learnable parameters.

### 4.8. MLP and LightGBM Baseline Models

In addition to the hypergraph model, two auxiliary models are trained using concatenated features:

$$x_{ijk}=\left[x_{d_{i}};x_{d_{j}};x_{c_{k}}\right]$$

where $x_{d_{i}}$, $x_{d_{j}}$, and $x_{c_{k}}$ are 512-dimensional vectors for drug 1, drug 2, and the cell line, respectively. The final concatenated feature dimension is 1536. The MLP consists of fully connected layers with dimensions 256, 128, and 64, each followed by batch normalization, ReLU activation, and dropout. LightGBM is trained with a binary objective, AUC metric, learning rate of 0.01, class imbalance handling, and early stopping. The ensemble weights were determined empirically during model development by evaluating multiple combinations of the three component weights on the validation set. Among the tested configurations, weights of 0.4, 0.3, and 0.3 for the hypergraph model, MLP, and LightGBM, respectively, achieved the best overall validation performance and were therefore selected for the final soft ensemble. The slightly higher weight assigned to the hypergraph branch is also consistent with its role as the principal higher-order interaction modeling component in SynerHyper. The test set was not involved in the selection of the ensemble weights.

### 4.9. Soft Ensemble Fusion

To improve predictive robustness, the final prediction score is obtained by soft fusion of the hypergraph model, MLP, and LightGBM predictions:

$${\hat{y}}_{final}=\omega_{HG}\cdot {\hat{y}}_{HG}+\omega_{\mathrm{MLP}}\cdot {\hat{y}}_{MLP}+\omega_{LGBM}\cdot {\hat{y}}_{LGBM}$$

with weights $\omega_{\mathrm{HG}}=0.4$, $\omega_{\mathrm{MLP}}=\omega_{\mathrm{LGBM}}=0.3$ (satisfying ∑ω=1). The final binary prediction is obtained by comparing against the optimal threshold $\tau^{*}$:

$${\hat{y}}_{final}^{\left(label\right)}=1\left[{\hat{y}}_{final}\geq \tau^{*}\right]$$

### 4.10. Training and Evaluation Protocol

To ensure an unbiased evaluation, the original dataset was first divided into training, validation, and test subsets before any data augmentation or parameter-dependent preprocessing. The three subsets contained 21,784, 6224, and 3113 observations, respectively. All preprocessing procedures requiring parameter estimation, including feature scaling and autoencoder training, were performed exclusively using the training subset. Data augmentation was subsequently applied only to the training subset, increasing the training data size from 21,784 to 36,411 observations. The validation and test subsets remained unchanged throughout model development.

The hypergraph model was trained using binary cross-entropy with logits loss and class weighting based on the ratio of negative to positive training samples. The Adam optimizer was used with weight decay. The validation set was used for model selection based on ROC-AUC. During training, test-set performance was monitored for descriptive purposes only and was not used for parameter optimization, ensemble-weight selection, or checkpoint determination. The final model checkpoint was selected according to validation performance and subsequently evaluated on the held-out test subset. During training, an optimal classification threshold was searched within the range of 0.30 to 0.65 with a step size of 0.05 according to validation F1-score. Model performance was assessed using ROC-AUC, average precision, F1-score, precision, recall, and confusion matrix analysis. Training curves for loss, AUC, and F1-score were generated to monitor model convergence.

## Figures and Tables

**Figure 1:**
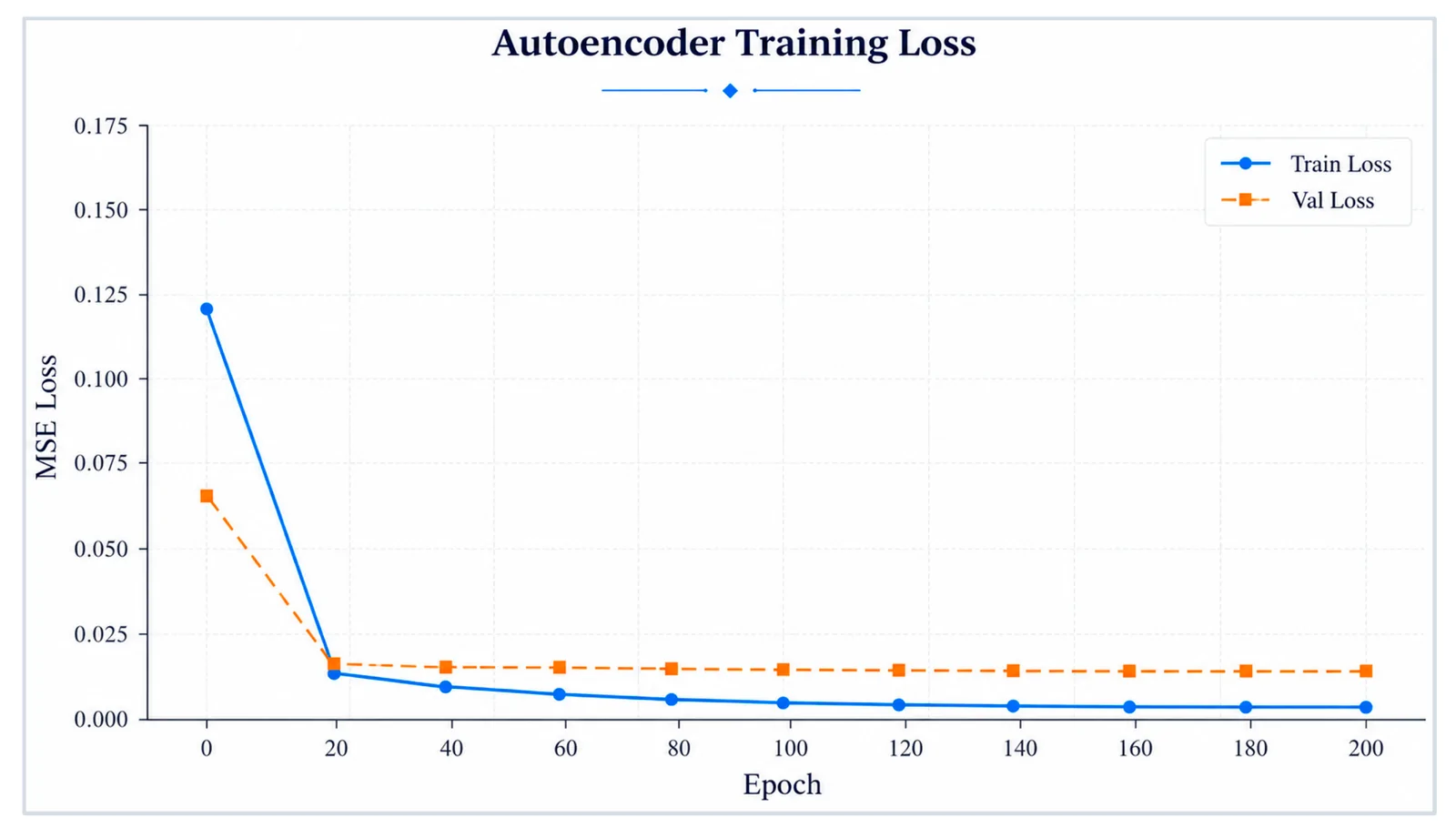
Autoencoder reconstruction performance during transcriptomic feature compression. Training and validation reconstruction loss curves are shown. The autoencoder was optimized using MSE loss to compress high-dimensional (4079-dim) transcriptomic profiles into 512-dimensional latent embeddings. Both training and validation losses decreased rapidly during early training and gradually stabilized, indicating effective convergence and stable representation learning. The low final reconstruction error suggests that the learned latent embeddings successfully preserved essential transcriptomic information while reducing feature dimensionality.

**Figure 2:**
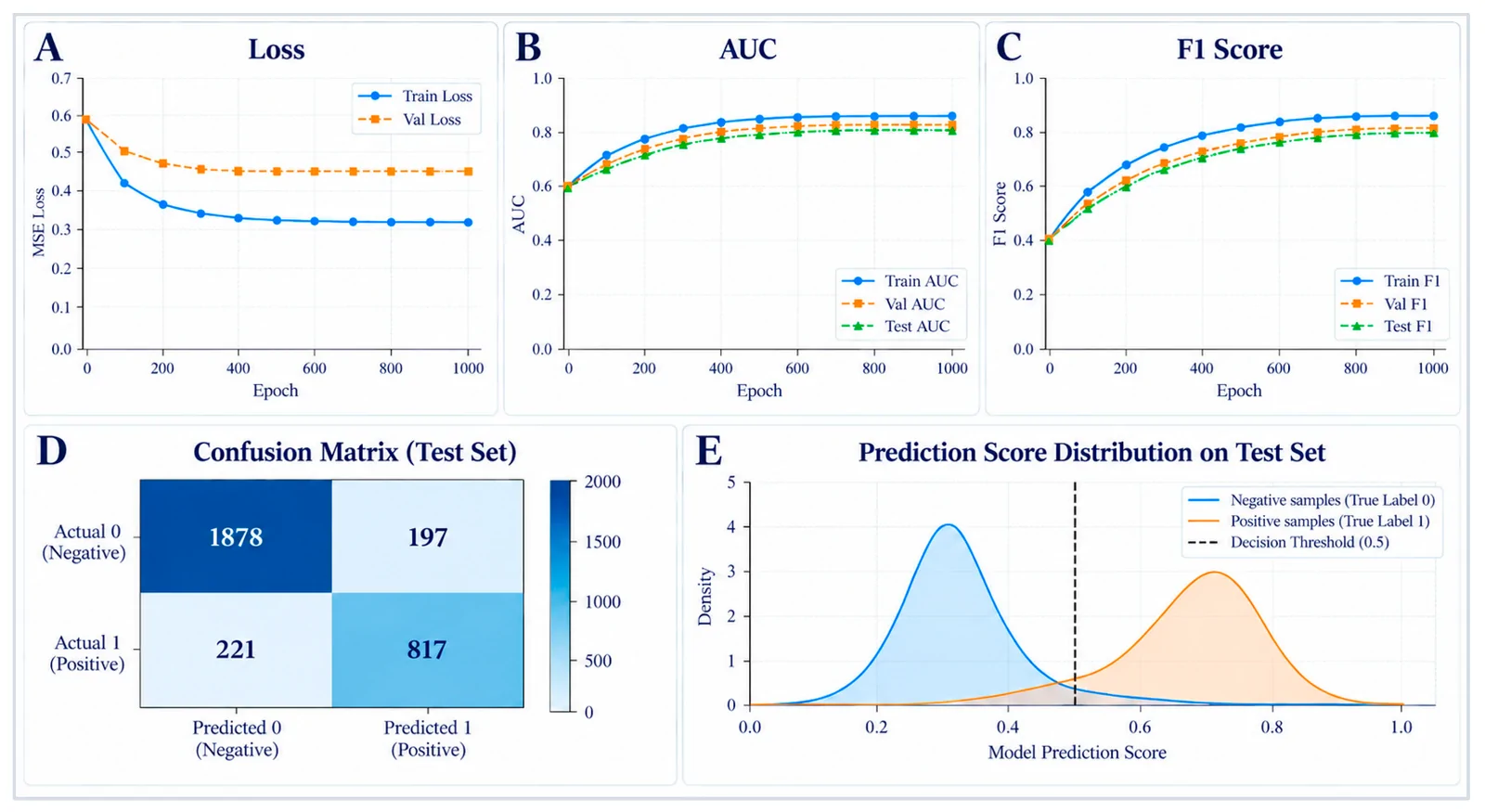
Predictive performance of the SynerHyper ensemble under the split-before-augmentation evaluation protocol. (**A**) Training and validation loss curves of the SynerHyper framework during model optimization. Both losses decreased progressively and gradually stabilized, indicating stable convergence. (**B**) AUC trajectories of the SynerHyper ensemble on the training, validation, and test subsets. The final checkpoint was selected exclusively according to validation-set performance. At the validation-selected checkpoint, the ensemble achieved a validation AUC of 0.815 and a corresponding test AUC of 0.808. (**C**) F1-score trajectories of the SynerHyper ensemble on the training, validation, and test subsets. (**D**) Confusion matrix of the SynerHyper ensemble on the test subset at the validation-selected checkpoint. The model correctly classified 1878 non-synergistic and 817 synergistic samples, with 197 false positives and 221 false negatives. (**E**) Distribution of ensemble prediction scores for non-synergistic and synergistic samples in the test subset. Synergistic samples generally received higher predicted probabilities, while partial overlap remained around the applied decision threshold.

**Figure 3:**
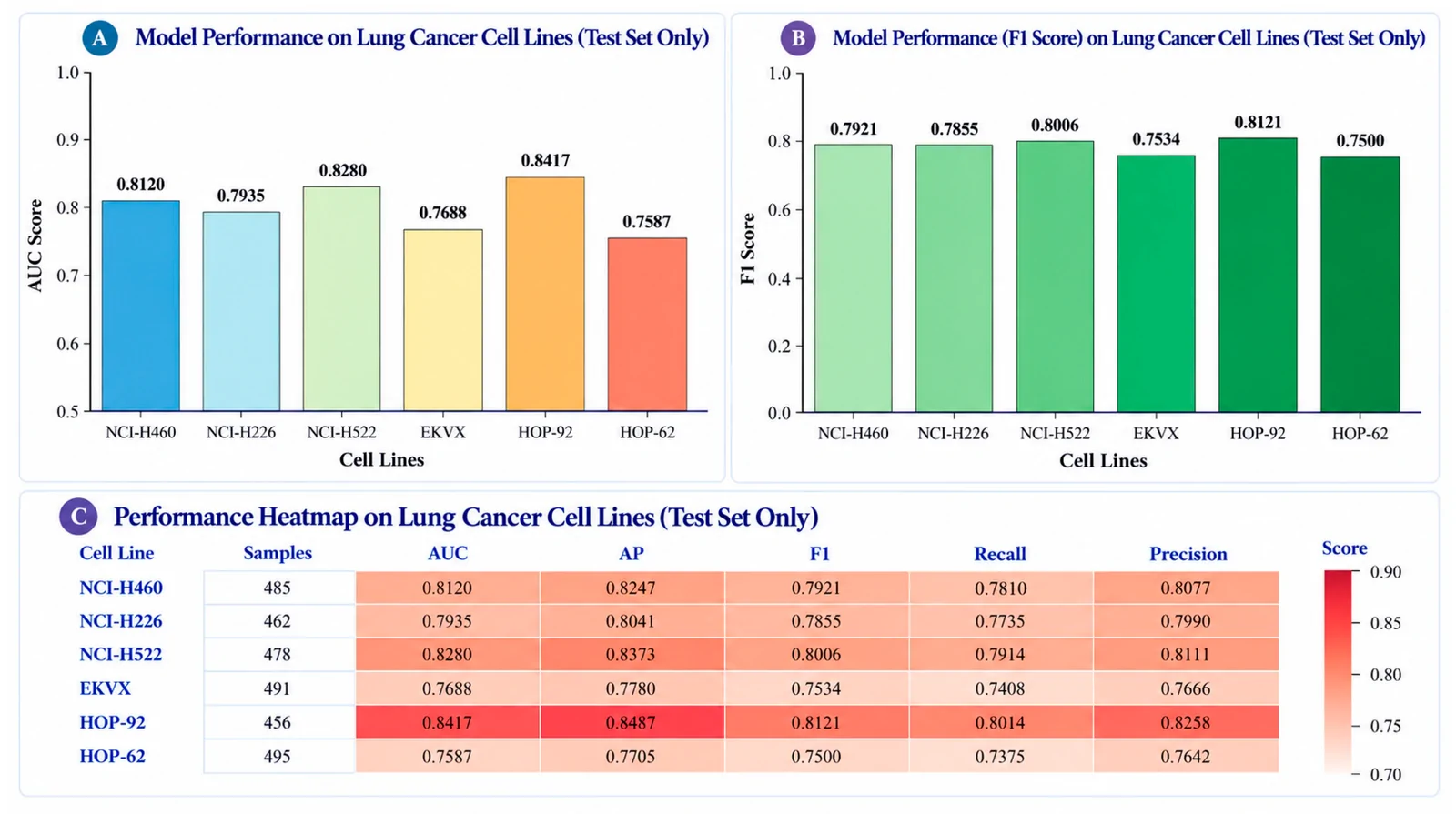
Cell-line-specific predictive performance of SynerHyper on the held-out test subset. (**A**) Test-set AUC values across six representative lung cancer cell lines. AUC values ranged from 0.759 to 0.842, with HOP-92 showing the highest performance (AUC = 0.842) and HOP-62 the lowest (AUC = 0.759). (**B**) Test-set F1 scores across the same six lung cancer cell lines. F1 scores ranged from 0.750 to 0.812, with HOP-92 achieving the highest F1 score (0.812) and HOP-62 the lowest (0.750). (**C**) Comprehensive heatmap summarizing the number of test samples and multiple performance metrics, including AUC, average precision (AP), F1 score, recall, and precision, for each cell line. The number of test samples per cell line ranged from 456 to 495. Recall values ranged from 0.738 to 0.801, precision from 0.764 to 0.826, and AP from 0.771 to 0.849, demonstrating measurable variation in predictive performance across different cellular contexts. These results represent cell-line-specific subgroup analyses within the held-out test subset rather than leave-cell-line-out generalization to completely unseen cell lines.

**Figure 4:**
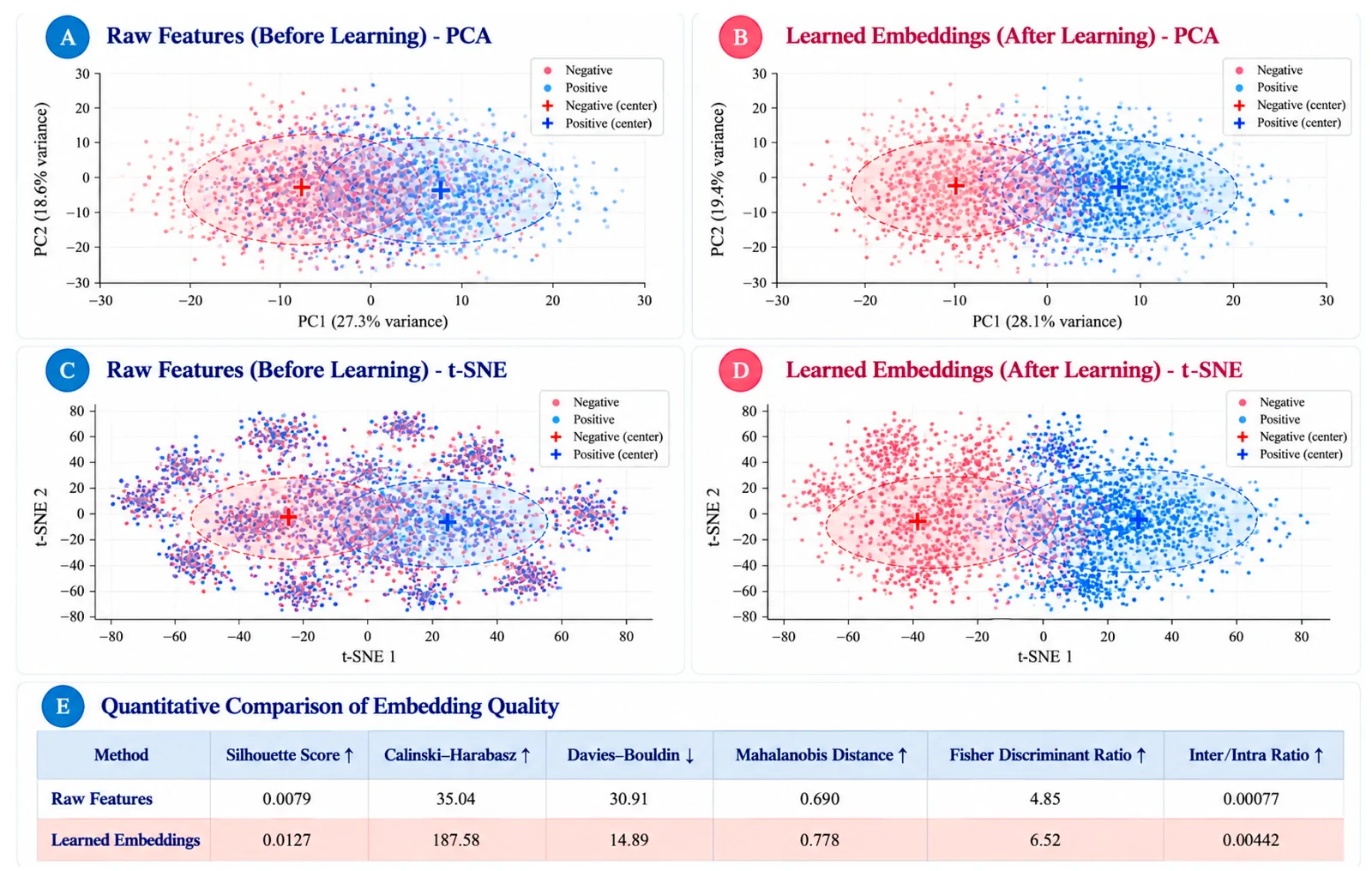
Visualization and quantitative assessment of feature-space organization before and after representation learning. (**A**) PCA projection of the raw multimodal features before representation learning. Synergistic and non-synergistic samples show substantial overlap, with only limited separation between the corresponding class centroids. (**B**) PCA projection of the learned hypergraph-level embeddings extracted from the trained SynerHyper framework. The class centroids become more distinguishable and the class distributions show improved directional organization, although considerable overlap remains. (**C**) t-SNE visualization of the raw multimodal features, showing extensive local mixing between synergistic and non-synergistic samples. (**D**) t-SNE visualization of the learned embeddings after training. Improved local organization is observed, while the two classes remain partially overlapping. Dashed ellipses indicate the 95% confidence regions of the corresponding classes, and cross markers denote class centroids. (**E**) Quantitative comparison of class-separation metrics between raw features and learned embeddings. The silhouette score increased from 0.0079 to 0.0127, the Calinski–Harabasz index from 35.04 to 187.58, the Mahalanobis distance from 0.690 to 0.778, the Fisher discriminant ratio from 4.85 to 6.52, and the inter-/intra-class distance ratio from 0.00077 to 0.00442, whereas the Davies–Bouldin index decreased from 30.91 to 14.89. Collectively, these changes indicate improved organization and class discrimination after representation learning, although the low absolute separation metrics and persistent visual overlap do not support complete class separation.

**Figure 5:**
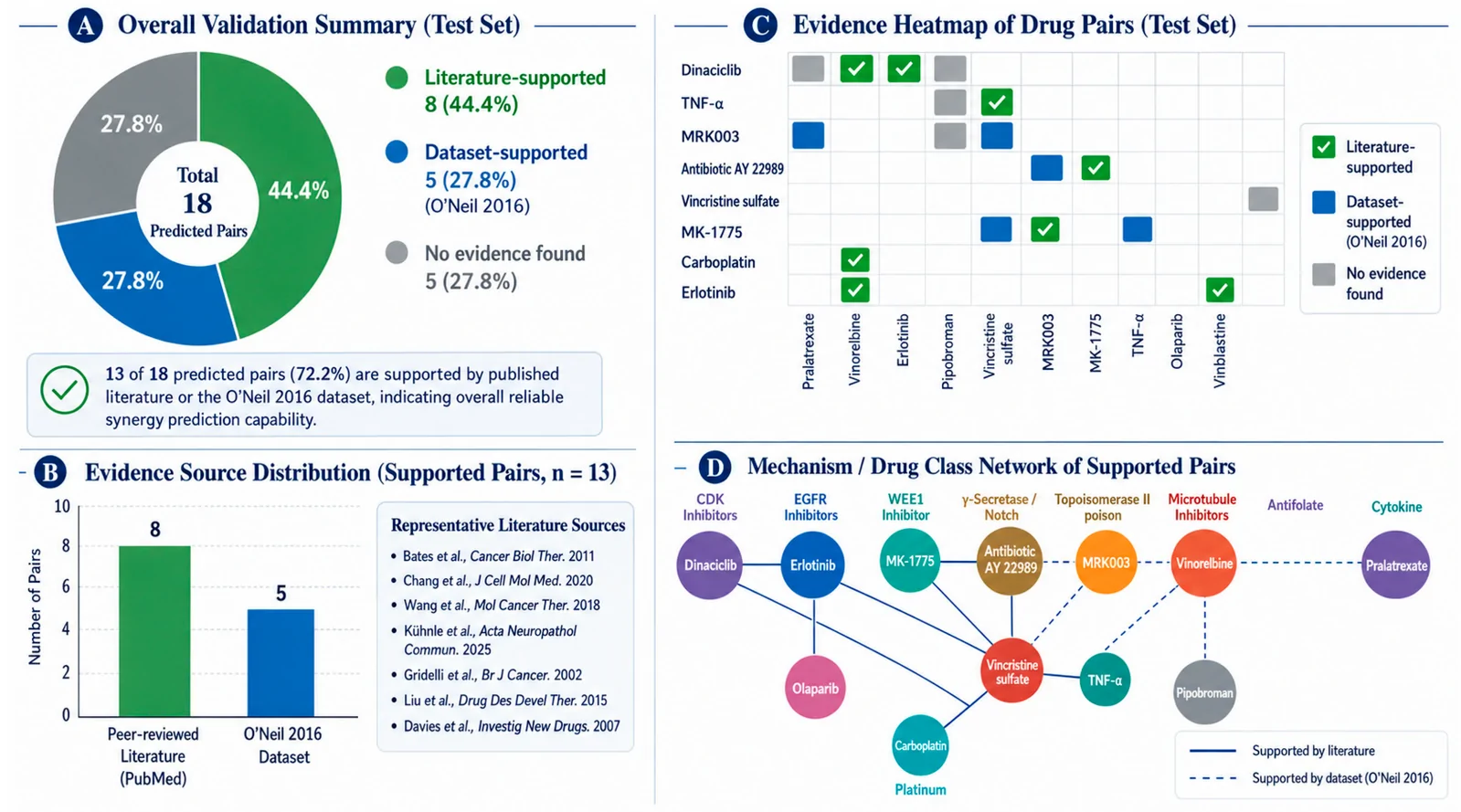
Retrospective evidence assessment and mechanistic analysis of selected high-ranking drug combinations. (**A**) Overall validation summary of the top predicted drug combinations. Among the 18 high-confidence synergistic drug pairs predicted by SynerHyper, 8 pairs (44.4%) were supported by previously published literature, 5 pairs (27.8%) were supported by the O’Neil 2016 drug synergy dataset, and 5 pairs (27.8%) lacked currently available supporting evidence. (**B**) Distribution of evidence sources among supported drug combinations. Most literature-supported combinations were supported by peer-reviewed studies indexed in PubMed, whereas 5 additional combinations were supported by the O’Neil 2016 dataset, providing additional retrospective support for the selected predictions. (**C**) Evidence heatmap of predicted drug combinations. Each cell represents a predicted synergistic drug pair. Green squares indicate literature-supported combinations, blue squares indicate combinations supported by the O’Neil 2016 dataset, and gray squares indicate combinations without currently available evidence. The heatmap shows that the majority of high-scoring predicted combinations are supported by literature or held-out database evidence. (**D**) Mechanism and drug-class interaction network of supported drug pairs. The identified synergistic combinations clustered into several biologically meaningful therapeutic modules, including cell-cycle and checkpoint regulation (CDK and WEE1 inhibition), receptor signaling inhibition (EGFR and PARP targeting), microtubule-targeting chemotherapy, and additional cross-class interactions involving antifolates, platinum agents, cytokine modulation, and DNA-damaging drugs. Dashed edges indicate support from the O’Neil dataset, whereas solid edges represent literature-supported interactions.

**Figure 6:**
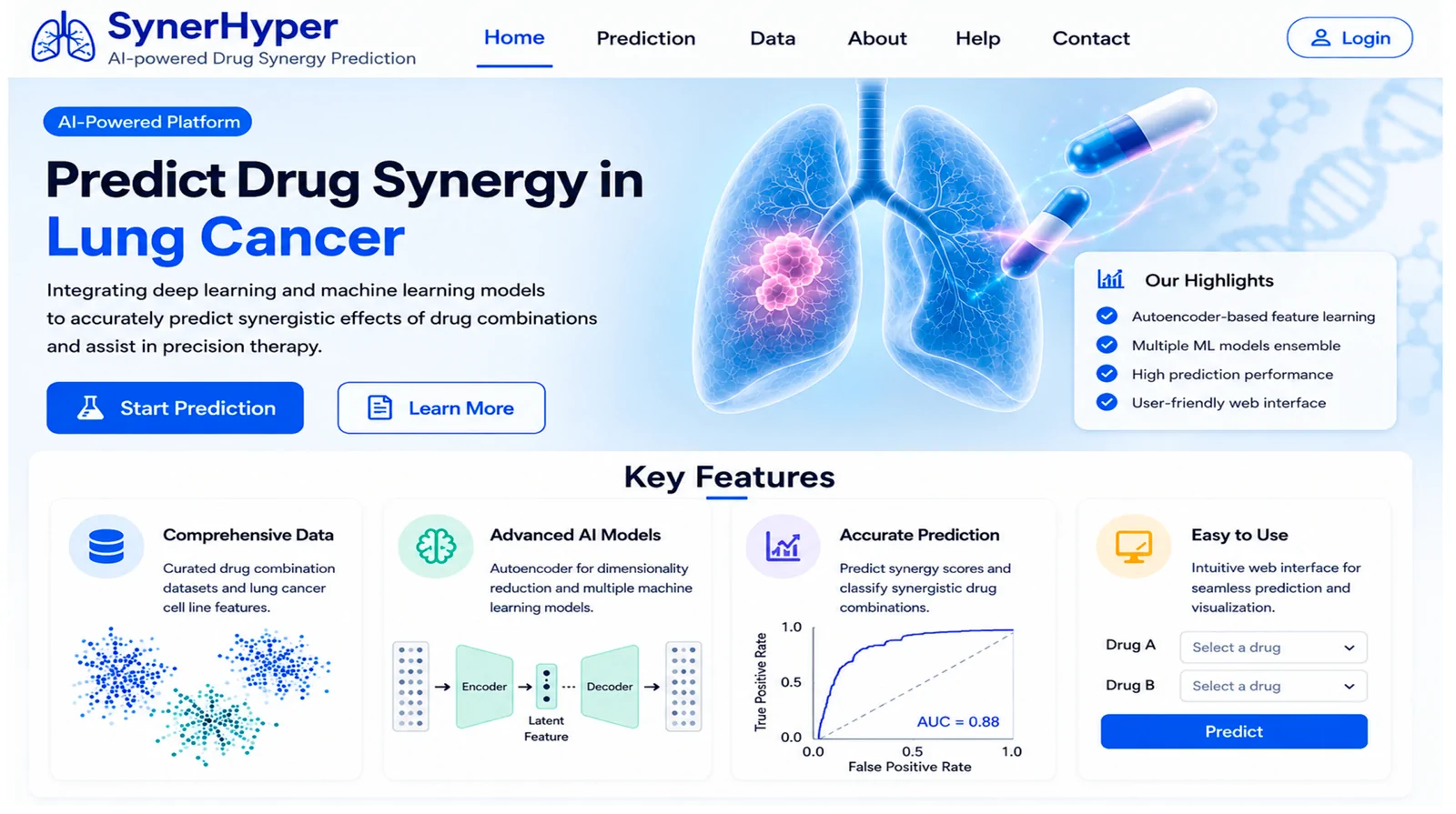
Web server interface of SynerHyper. The web server provides an interactive platform for lung cancer drug synergy prediction. Users can input candidate drug combinations through a user-friendly interface and obtain real-time prediction results. The platform integrates transcriptomic representation learning, hypergraph neural modeling, and ensemble prediction modules within an accessible web-based environment.

**Table 1:** Performance comparison of representative drug-synergy prediction models on the lung cancer dataset.

Model	Architecture Type	Best AUC
DTSyn	Transformer-based	0.690
DeepSynergy	MLP-based	0.750
DeepDDS (GAT)	Graph Attention Network	0.714
DeepDDS (GCN)	Graph Convolution Network	0.792
AttenSyn	Attention-based	0.780
**SynerHyper**	**Hypergraph + Uni-Mol + Ensemble**	**0.808**

Note: The baseline AUC values were calculated in the present study by applying the corresponding existing drug-synergy prediction models to the lung cancer dataset; they were not taken from the original publications. The baseline models were used as general anticancer drug-synergy predictors without lung cancer-specific retraining or optimization.

**Table 2:** Ablation study of SynerHyper under the revised split-before-augmentation evaluation protocol.

Model Variant	Description	Test AUC
**Full model**	**Complete framework**	**0.808**
w/o Hypergraph	Remove HGN branch	0.722
w/o MLP	Remove MLP branch	0.780
w/o Ensemble Fusion	Direct concatenation	0.792
w/o Uni-Mol	Replace with traditional fingerprints	0.716
w/o Autoencoder	Use raw transcriptomics	0.736
Hypergraph only	Only HGN branch	0.773
MLP only	Only MLP branch	0.668

## Data Availability

The raw drug combination datasets (DrugComb and GDSC2) supporting this study are publicly accessible at http://drugcombdb.denglab.org/main, accessed on 17 September 2026 and https://gdsc-combinations.depmap.sanger.ac.uk/, accessed on 17 September 2026, respectively. The processed lung cancer-specific subset, source code, trained SynerHyper models, and online web server program are available at https://github.com/sunchuance/SynerHyperr, accessed on 17 September 2026. All supplementary analysis files and figure generation scripts are deposited in the same repository.
